# Natural Products for Liver Diseases: Basic, Clinical, and Translational Research

**DOI:** 10.1155/2012/794343

**Published:** 2012-12-31

**Authors:** Chang-Quan Ling, Jen-Hwey Chiu, Byeongsang Oh, William C. S. Cho

**Affiliations:** ^1^Department of Traditional Chinese Medicine, Changhai Hospital Affiliated to the Second Military Medical University, Shanghai 200433, China; ^2^Institute of Traditional Medicine, National Yang-Ming University, Taipei, 11221, Taiwan; ^3^Sydney Medical School, University of Sydney, Fisher Road, Sydney, NSW 2006, Australia; ^4^Department of Clinical Oncology, Queen Elizabeth Hospital, Kowloon, Hong Kong

Liver diseases are important causes of human morbidity and mortality in the world. The incidence of liver diseases is rising due to the widespread of hepatitis and alcoholism. They may be caused by infection, injury, exposure to drugs or toxic compounds, autoimmunity, or genetic defect that leads to the deposition of harmful substances. Liver damage is likely to play a role in inflammation, scarring, obstructions, cirrhosis, liver failure, and even liver cancer.

The use of herbal medicines can be traced back several thousand years ago in ancient China. A large volume of evidences have shown that many natural products are available as chemoprotective agents against common liver diseases, such as hepatitis, cirrhosis, liver cancer, fatty liver diseases, and gallstones. This special issue aims to gather updated progress in this important area.

The papers in this special issue cover a wide range of topics, including a study of the anti-hepatitis B activity of 3,4-*O*-dicaffeoylquinic acid isolated from *Laggera alata *using the D-galactosamine-induced hepatocyte damage model and hepatitis B virus transgenic mice. *In vitro *results showed that the compound could markedly inhibit the production of HBsAg and HBeAg, whereas *in vivo *results indicated that the test compound significantly inhibited HBsAg production and increased heme oxygenase-1 expression in hepatitis B virus transgenic mice.

On the other hand, the study of *Graptopetalum paraguayense* showed cytotoxicity toward hepatic stellate cells. Microarray profiling indicated that the expression of most metabolism- and cell growth and/or maintenance-related genes recovered to near normal levels following *G. paraguayense* treatment as classified by gene ontology and laser scanning microscope analysis. A publicly accessible website was further established to present data of the identified genes, including 44 necroinflammation-related and 62 fibrosis-related genes. These might provide useful insight into the molecular mechanisms underlying liver damage and potential targets for the development of therapeutic drugs.

Another study demonstrated that the ethanolic extracts of* Cassia sophera *Linn. leaves showed hepatoprotective activity against carbon tetrachloride-induced hepatic damage in rats. Their study suggested that this possible activity might be due to the presence of flavonoids in the extracts.

*Elephantopus scaber *has been traditionally used as liver tonic. However, its protective effect on liver damage is still unclear. In the study of the total phenolic and flavonoid content of *E. scaber *ethanol extract, low concentration of *E. scaber *was able to reduce serum biochemical profiles and fat accumulation in the liver of alcohol-induced liver damage mice, while high concentration of *E. scaber* was able to revert the liver damage without possessing any oral acute toxicity on mice.

Chinese herbal medicines usually treat disease with a prescription of several herbs mixing together. It has been demonstrated that the antifibrotic properties of a Chinese herbal decoction Danggui Buxue Tang in a rat model of liver fibrosis were related to its ability to inhibit angiogenesis. This antiangiogenic mechanism was associated with the improvement of oxidative stress, the expression and signaling regulation of angiogenic factors, and especially the modulation of hypoxia inducible factor-1*α* in fibrotic livers.

A number of anticancer herbal medicines have demonstrated anticancer effects [1]. Hepatocellular carcinoma (HCC) is always hard to treat; anoikis has been recognized as a potential target for anticancer therapy. *Polygonum cuspidatum* is a frequently used Chinese herb in the treatment of HCC. A study in this special issue showed an inhibitory effect of *P. cuspidatum* extract in HCC cells proliferation in a dose- and time-dependent manner. *P. cuspidatum* extract also inhibited the anchorage-independent growth of HCC cells in soft agar by inducing caspase-mediated anoikis in HCC cells which might relate to reactive oxygen species generation and focal adhesion kinase downregulation. Their study has provided new insight into the application of Chinese herbs for HCC treatment.

On the other hand, the root of *Actinidia valvata* has been widely used in the treatment of HCC. A report in this special issue demonstrated that the total saponin of *A. valvata* could effectively inhibit HCC growth and metastasis *in vivo*, inhibit the formation of microvessel, downregulate the expressions of vascular endothelial growth factor and basic fibroblast growth factor, and retrain angiogenesis of hepatoma.

In the omics era, a number of studies use omics technologies to study herbal medicines [2]. A ^1^H nuclear magnetic resonance-based metabonomic approach was proposed to explore the biochemical characteristics of Yang deficiency syndrome in HCC based on serum metabolic profiling. The decreased intensities of metabolites (including low density lipoprotein/very-low density lipoprotein, isoleucine, lactate, lipids, choline, and glucose/sugars) in serum might be the distinctive metabolic variations of Yang deficiency syndrome patients with HCC. These metabolites might be potential biomarkers for the diagnosis of Yang deficiency syndrome in HCC.

Finally, a review article entitled “Herbal products: benefits, limits, and applications in chronic liver disease” described various examples of herbal products on pharmacokinetics, biological, and beneficial effects in metabolic, alcoholic, and viral hepatitis. The authors also pointed out that the lack of randomized, placebo-controlled clinical trials might be the main limitation of the applicability of complementary and alternative medicine.

We envision that this special issue will arise more interest in the field of hepatology, and more exciting investigations on liver diseases by herbal medicine will be conducted.

*Chang-Quan Ling* *Chang-Quan Ling* *Jen-Hwey Chiu* *Jen-Hwey Chiu* *Byeongsang Oh* *Byeongsang Oh* *William C. S. Cho* *William C. S. Cho*
